# Molecular Identification and Characterization of Probiotic *Bacillus* Species with the Ability to Control *Vibrio* spp. in Wild Fish Intestines and Sponges from the Vietnam Sea

**DOI:** 10.3390/microorganisms9091927

**Published:** 2021-09-10

**Authors:** Khanh Minh Chau, Thi Thu Hao Van, Dong Van Quyen, Hung Dinh Le, Trinh Hoai Thi Phan, Ngoc Duy Thi Ngo, Trang Dieu Thi Vo, Trung Thanh Dinh, Hoa Thi Le, Huynh Hoang Nhu Khanh

**Affiliations:** 1 Nha Trang Institute of Technology Research and Application, Vietnam Academy of Science and Technology, 02 Hung Vuong, Loc Tho, Nha Trang City 57000, Vietnam; minhkhanhchau@gmail.com (K.M.C.); ledinhhungims@yahoo.co.uk (H.D.L.); phanhoaitrinh84@gmail.com (T.H.T.P.); duyngoc@nitra.vast.vn (N.D.T.N.); vtrang47@gmail.com (T.D.T.V.); dinhthanhtrung410@gmail.com (T.T.D.); lethihoa@nitra.vast.vn (H.T.L.); 2 School of Science, RMIT University, Bundoora, VIC 3083, Australia; thithuhao.van@rmit.edu.au; 3 Institute of Biotechnology, Vietnam Academy of Science and Technology, 18 Hoang Quoc Viet, Cau Giay, Ha Noi City 100000, Vietnam; dvquyen@ibt.ac.vn

**Keywords:** anti-*Vibrio* activity, polyketide synthase (PKS), non-ribosomal peptide synthetase (NRPS), bacteriocin, aquaculture

## Abstract

Vibriosis in farmed animals is a serious threat to aquaculture worldwide. Using probiotics and anti-*Vibrio* antimicrobial substances in aquaculture systems can be a means of preventing *Vibrio* infections. Therefore, we aimed to characterize and compare 16 potential anti-*Vibrio* probiotics (Vi+) isolated from marine sponges and fish intestines collected from the Vietnam Sea, as well as an anti-*Vibrio* bacteriocin to fully explore their application potentials. 16S rRNA sequencing confirmed all Vi+ to be *Bacillus* species with different strain variants across two sample types. An obvious antimicrobial spectrum toward Gram-negative bacteria was observed from intestinal Vi+ compared to sponge-associated Vi+. The reason was the higher gene frequency of two antimicrobial compounds, non-ribosomal peptides (NRPS) and polyketide type-I (PKS-I) from intestinal Vi+ (66.7%) than sponge-associated Vi+ (14.3% and 0%, respectively). Additionally, a three-step procedure was performed to purify an anti-*Vibrio* bacteriocin produced by *B. methylotrophicus* NTBD1, including (i) solvent extraction of bacteriocin from cells, (ii) hydrophobic interaction chromatography, and (iii) reverse-phase HPLC. The bacteriocin had a molecular weight of ~2–5 kDa, was sensitive to proteolysis and thermally stable, and showed a broad antimicrobial spectrum, all of which are essential properties for promising feed additives. This study provides necessary information of the potential of probiotic *Bacillus* species with anti-*Vibrio* antimicrobial properties to study their further use in sustainable aquaculture.

## 1. Introduction

Aquaculture is of great economic importance around the world, including in Vietnam. However, vibriosis in commercial fish and shrimp, due to infection by *Vibrio* species, causes failures in aquaculture, leading to an economic burden and social losses. *Vibrio* pathogens are well known as Gram-negative and saltwater-borne bacteria. *Vibrio* species, which are commonly present in the gastro-intestinal tract of aquatic animals, may produce large amounts of toxins that cause many acute illnesses. For instance, V. *parahaemolyticus* causes acute hepatopancreatic vibriosis necrosis disease (AHPND) in shrimp, with a mortality rate of up to 100%, and other *Vibrio* species (i.e., *V. alginolyticus*, *V. harveyi*, *V. cholera*, and *V. anguillarum*) have a significant effect on farmed shrimp, fish, crustaceans, and lobster [1]. It is noteworthy that reports have documented a rise in antibiotic-resistant *Vibrio* in aquaculture farms around the world due to the overuse of antibiotics for treatment. Many outbreaks of *V*. *parahaemolyticus* AHPND were recorded in the Mekong Delta, which is the largest seafood production area in Vietnam [2].

For sustaining aquaculture and the ecosystem, the elimination of antibiotic use and alternative promotion of probiotic use for the prevention of *Vibrio* infection in farming are urgently required. Compared with the terrestrial probiotic strains, the similar growth conditions of *Vibrio* and marine bacteria ensure the probiotic properties of marine anti-*Vibrio* bacteria (Vi+) and the stability of their antimicrobial molecules under saltwater aquaculture conditions. In recent years, the literature has reviewed a number of bacterial genera (i.e., *Vibrio*, *Bacillus*, *Proteus*, and *Streptomyces*) from the marine environment possessing anti-*Vibrio* activity. For instance, *B. subtilis* AQAHBS001, isolated from the Pacific white shrimp gut in Thailand, inhibited the growth of many *V. parahaemolyticus* AHPND strains [3]. Many microbial strains, including eleven *Vibrionaceae*, three coryneform, and one *Bacillus* NM 12, isolated from fish intestines in the Japan Sea were found to display remarkable activity against *V. vulnificus* [4]. In the Vietnam Sea, several Vi+ strains (belonging to the genera *Proteus*, *Bacillus*, and *Enterococcus*) were isolated from fish and lobster intestines collected in the Nha Trang Sea region and showed significant activity against *V. owensii* and *V. parahaemolyticus* [5]. Some antimicrobial substances produced by Vi+ have been purified and identified as common secondary metabolites (polyketides) or small peptidal structures (commonly <10 kDa), such as bacteriocins or non-ribosomal peptides (like lipopeptides). For instance, a lipopeptide produced by *B. amyloliquefaciens* M1 displayed inhibitory activity toward many multidrug-resistant *Vibrio* species [6]. The production of anti-*Vibrio* bacteriocins is frequently involved in *Bacillus* and *Vibrio* species [4,7,8]. These antimicrobial peptides are products of ribosomal biosynthetic pathway for bacteriocins or the non-ribosomal biosynthetic pathway for polyketides and lipopeptides and offer great potential as promising antimicrobial agents for use in the food industry, agriculture, aquaculture, and pharmaceuticals [9].

The Vietnam Sea has unique environmental parameters compared to other marine ecosystems, promising to be a valuable source of untapped antimicrobial substances. However, studies on the Vietnam Sea’s bacterial community, its potential activity against *Vibrio* sp., and the characterization of Vi+, are limited in the literature. In addition, the intestines of aquatic animals are likely a major isolation source of Vi+, although Vi+ is also present in sponges, which are the richest source of novel and diverse antimicrobial substances [10]. So far, it is still unknown whether the Vi+ across the two sample types is different in antimicrobial activity or not. There is an abundance of different marine sponge and fish species in Vietnam Sea, therefore it is of great value to identify species of Vi+ across two sources. Previously, we obtained a collection of anti-*Vibrio* probiotics isolated from marine sponges and wild fish intestines collected from the Vietnam Sea. This study aims to (1) further identify species of Vi+ across two isolated sources, (2) evaluate and compare the in vitro antimicrobial activity of these probiotics against a wide range of aquaculture, foodborne, and clinical or livestock pathogens, (3) give a preliminary explanation for the differences at a gene level, and (4) extract and purify a bacteriocin-like compound produced by an intestinal *B. methylotrophicus* NTBD1 for the characterization. The results obtained from the analysis can tell us whether these Vi+ are effective biocontrol probiotics for future aquaculture use.

## 2. Materials and Methods

### 2.1. Strain Collection

**Figure 1:**
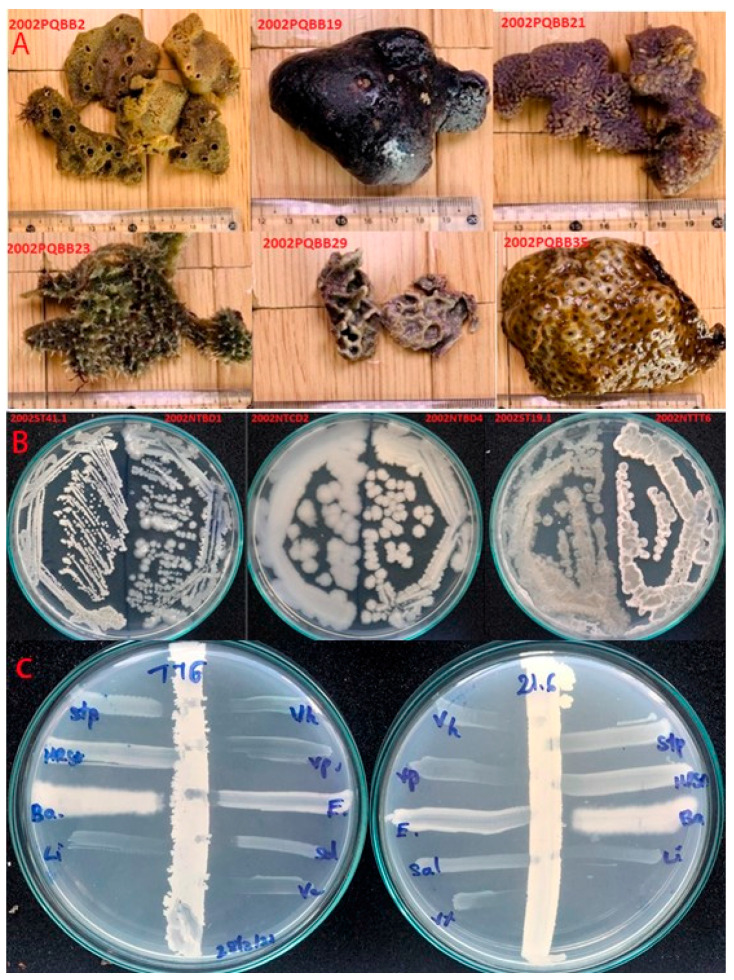
Basic information of isolated Vi+. (**A**) Pictures of marine sponges used for isolation of the sponge-associated Vi+; (**B**) Vi+ appeared to be diverse in colony morphology; (**C**) demonstration of the cross-streak assay for evaluating the antimicrobial spectra of isolated Vi+ from an intestinal Vi+ NTTT6 and a sponge-associated Vi+ PQBB21.6. Reference strains included *V. harveyi* (Vh), *V*. *parahaemolyticus* (Vp), *E. coli* (E), *S. typhimurium* (Sa), *S. aureus* (Stp), methicillin-resistant *Staphylococcus aureus* (MRSA), *B*. *cereus* (Ba), and *L. monocytogenes* (Li).

The sixteen marine bacteria showing the best performance in terms of antimicrobial activity toward *Vibrio parahaemolyticus* (Vi+) were selected for the analysis. They were stored at −80 °C in the Marine Microbial Culture Collection at Nha Trang Institute of Technology Research and Application, Vietnam Academy of Science and Technology. Of these, seven Vi+ were isolated from seven marine sponges collected in the Phu Quy Sea region (10°29′ N, 108°57′ E; 10°30′ N, 108°56′ E) by scuba diving at a water depth of 8–15 m; nine bacteria were isolated from five wild-caught fish and shrimp collected in the Nha Trang Sea region (12°14′ N, 109°11′ E). The sponge species are not yet identified; photographs are given in Figure 1A, while the wild fish and shrimp were identified as *Harpiosquilla* sp (TT), *Penaeus latisulcatus* (TB), *Aluterus* sp. (BD), *Priacanthus* sp. (ST), and *Nemipterus japonicas* (CD). Isolated Vi+ were sub-cultured from −80 °C stock onto Mueller–Hinton agar (Oxoid Ltd., Cambridge, UK) and incubated at 30 °C for overnight. The cultures displayed diverse colony morphology (Figure 1B) after incubation.

### 2.2. Screening of Vi+ Strains for Antimicrobial Activity toward Foodborne and Clinically Relevant Pathogens

The Vi+ were subjected to an agar-based cross-streak assay to evaluate the antimicrobial spectra. Nine microbes were used as reference bacteria in the cross-streak assays: *Vibrio parahaemolyticus* NMMCI01, *Vibrio harveyi* NMMCI02, *Salmonella typhimurium* ATCC6994, *Escherichia coli* ATCC25922, *Bacillus cereus* ATCC 11778, *Staphylococcus aureus* ATCC 25923, *Listeria monocytogenes* ATCC 19111, *Candida albicans* ATCC 10231, and methicillin-resistant *Staphylococcus aureus* (MRSA). Two *Vibrio* indicators were isolated from local infected shrimp, provided by the Research Institute for Aquaculture no. 3 (Vietnam). The majority of reference strains was sub-cultured from −80 °C stock onto Mueller–Hinton agar (Oxoid Ltd., Cambridge, UK), while *Vibrio* species were sub-cultured onto thiosulfate–citrate–bile salt–sucrose agar (TCBS) (Oxoid Ltd., Cambridge, UK) or Mueller–Hinton agar (MHA) supplemented with 3% NaCl.

In this study, a cross-streak assay (Figure 1C) was used for screening the antimicrobial activity of Vi+ due to its greater rapidness and effectiveness than other methods (i.e., the well diffusion method) [11]. Briefly, a Vi+ colony on a plate was picked up with a sterile tampon and drawn in a vertical line on lab-prepared marine agar plates (LPMA) (1 g/L glucose, 5 g/L peptone, 2 g/L yeast extract, 0.05 g/L MgSO_4_, 0.1 g/L, K_2_HPO_4_, 50% natural seawater, 50% distilled water), and then incubated at 30 °C overnight. Each reference strain was picked by a separate tampon and drawn in a horizontal line toward the Vi+ biomass, then incubated at 30 °C overnight to grow the reference strains. Antimicrobial activity was identified by measuring the inhibition distance (mm) between the biomass of reference strains and Vi+.

### 2.3. Extraction of Genomic DNA and Amplification of 16S rRNA Sequence

Species identification of 16 Vi+ was conducted by the 16S rRNA sequencing method. Bacterial genomic DNA was extracted and used as templates for the amplification of the 16S rRNA gene using a primer pair (27F/1492R), as described earlier [12]. A PCR reaction consisted of 12.5 μL of MyTaq™ mix (Bioline Reagents Limited, London, UK), 1 μL of each forward and reverse primer (10 pmol), 1 μL of 100 ng/μL gDNA, and distilled water added to obtain a total volume of 25 μL. PCR amplification consisted of an annealing step at 95 °C for 10 min, 30 cycles of (95 °C for 30 s, 48 °C for 30 s, 72 °C for 90 s), and a final extension at 72 °C for 10 min.

### 2.4. Amplification of Nonribosomal Peptide and Polyketide Biosynthetic Gene Clusters

To explain antimicrobial spectra, all of the 16 Vi+ were checked for antimicrobial biosynthetic gene clusters relating to the production of non-ribosomal peptides, polyketide type I (PKS-I), and polyketide type II (PKS-II). These antimicrobial substances have shown activity against a wide range of other bacteria including *Vibrio* species [6,13,14]. A PCR reaction was performed to amplify conserved gene regions within the genes coding non-ribosomal peptide synthetase (NRPS), polyketide type I synthetase (PKS-I), and polyketide synthetase (PKS-II) enzymes. Three primer pairs, as previously reported [15], were employed: A3F (5′-GCSTACSYSATSTACACSTCSGG-3′)/A7R (5′-SAS GTCVCCSGTSCGGTAS-3′) for amplification of a ~700–800 bp *NRPS* gene product, MDPQQR_f (5′-ATGGATCCGCAGCAACG-3′)/HGTGT_r (5′-AGTGCCAGTGCCGTG-3′) for a ~700–800 bp *PKS-I* gene product, and PF6 (5′-TSGCSTGCTTGGAYGCSATC)/PR6 (5′-TGGAANCCGCCGAABCCGCT-3′) for a *PKS-II* gene product of ~600–700 bp. In the primer sequences, B = C/T/G, I = Inosine, K = G/T, N = A/G/C/T, and S = G/C, Y = C/T.

The PCR cycle with a (A3F/A7R) primer consisted of a denaturing step at 95 °C for 5 min, 35 cycles of (95 °C for 30 s, 59 °C for 2 min, 72 °C for 1.5 min), and a final extension of 10 min at 72 °C. The PCR cycle with (MDPQQR_f/HGTGT_r) consisted of 5 min at 95 °C, 30 cycles of (1 min at 95 °C, 30 s at 53 °C, 72 °C for 1.5 min), and 1 min at 72 °C. The cycle with primer (PF6/PR6) consisted of 96 °C for 5 min, 30 cycles of (96 °C for 1 min and 59 °C for 1 min and 72 °C for 1.5 min), and 10 min at 72 °C. Genomic DNA of a *B. amyloliquefaciens* #11 in the Marine Microbial Culture Collection was used as a positive control due to carrying the *PKS-I* and *NRPS* genes.

### 2.5. Sequencing of 16S rRNA Products and Antimicrobial Gene Products

PCR products were run in 1.2% (*w/v*) agarose gel along with hyperLadder™ 100 bp (Bioline Reagents Limited, London, UK) for checking the size, then purified and sent to the 1st BASE DNA sequencing company (Axil Scientific Pte Ltd., Singapore Science Park II, Singapore) for Sanger sequencing of one side using a 27F primer (for the 16S rRNA products) or MDPQQR_f primer (for PKS-I gene product). The raw reads were trimmed unqualified nucleotides at two ends, and we searched against a nonredundant database (NCBI) to identify the closely homologous sequences.

### 2.6. Effect of Cultivation Time on Anti-Vibrio Bacteriocin Production

*B. methylotrophicus* NTBD01 was selected for the purification of bacteriocin as the anti-*Vibrio* activity of the culture appeared to be sensitive to proteolysis using protease E (Sigma-Aldrich, MO, USA).

To optimize cultivation times for high-yield production of bacteriocin, the antimicrobial activity of cultures obtained at different cultivation times were evaluated. Overnight bacterial culture (1%) was inoculated in 100 mL of lab-prepared marine broth (LPMB), shaken at 120 rpm at 30 °C. Five milliliters of the culture were collected at cultivation times of 3 h, 6 h, 9 h, 12 h, 24 h, 27 h, 30 h, 33 h, and 48 h. The optical density of cultures was measured at a wavelength of 660 nm (OD_600_), then the cultures were centrifuged at 4500× *g* for 20 min, and the obtained supernatant we filtered through 0.45-µm polyether sulfone (PES) membranes to obtain the cell-free system (CFS). The antimicrobial activity of CFSs was evaluated using a well diffusion assay against *V*. *parahaemolyticus* and *L. monocytogenes* [11]. A Mueller–Hinton agar plate (1.5% agar, supplemented with 3% NaCl) was spread with saline buffer containing each reference strain (~10^6^ CFU/mL) using a cotton swab. Holes with a diameter of 6 mm were made and 80–100 µL of CFS were introduced to holes and left until complete diffusion. The antimicrobial activity was identified by measuring the inhibition diameter (mm) around the holes. All of the active cultures were obtained and then incubated with 1 mg/mL protease E (Sigma-Aldrich) at 37 °C for 1 h to check for the presence of a proteolytic sensitive bacteriocin-like compound.

### 2.7. Purification of Bacteriocin Produced by B. methylotrophicus NTBD01

Ten milliliters of the starter culture (1%) of *B. methylotrophicus* NTBD01 were inoculated in 1 L of LPMB culture, shaken at 120 rpm at 30 °C for the optimal time. To extract the bacteriocin, cells were collected by centrifugation at 4500× *g* for 20 min and stirred into 200 mL isopropanol (70%) at pH 2 (adjusted with HCl) at 5 °C overnight, as in an earlier report [16]. A crude bacteriocin extract (CRE) was prepared by centrifugation of the mixture at 4500× *g* for 20 min to remove cells, filtration of the solvent through a PES membrane (0.45 µm pore size), and concentration with the aid of a rotary evaporator (Buchi Corporation, New Castle, DE, USA) to remove the solvent.

The CRE was subsequently applied to a Sep-Pak C18 cartridge (WAT036925, Waters Corporation, Milford, MA, USA) equilibrated with 5% methanol in water (+0.1% trifluoroacetic acid (TFA)). The cartridge was washed with 5% methanol (+0.1% TFA), eluted with an increasing concentration of methanol (+0.1% TFA) of 30%, 50%, 70%, and 100%, and a final elution with 70% isopropanol at pH 2. Each step was carried out with three column volumes. The fractions were collected and tested against *V*. *parahaemolyticus* as a reference strain. The active fraction was lyophilized, dissolved in a minimum volume of water, and introduced to a reverse-phase, high-pressure liquid chromatography (RP-HPLC) system (Shimadzu Corporation, Kyoto, Japan). About 100 µL of active fraction were injected into an analytical C18 column (Shim-pack GIST column, 150 mm × 3.0 mm, 3 μm) equilibrated with 5% solvent A (acetonitrile, +0.1% TFA) in solvent B (water, +0.1% TFA). Elution was carried out with a gradient from 5% to 100% solvent A for 55 min, at a flow rate of 1 mL min^−1^, detected by UV detector wavelengths of 220 nm and 280 nm, and collected manually at intervals of 2 min. All of the obtained elutes were tested for anti-*Vibrio* activity. The active fraction was lyophilized and dissolved in the minimum amount of water. About 100 µL of the sample were again injected into a C18 column equilibrated with 40% solution A. The elution was performed with a gradient concentration of 40%–60% solution A for 50 min. Every protein peak was collected manually by the peak-picking method with the aid of a UV detector at a wavelength of 220 nm and then tested for the bioactivity. To obtain the retention time of bacteriocin, 20 µL of the active fraction were reinjected into the C18 column under the performance of a gradient from 5% to 100% solvent A for 55 min, as in the first cycle.

### 2.8. Estimation of Bacteriocin’s Molecular Mass by TRICINE Sodium Dodecyl Sulfate-Polyacrylamide Gel Electrophoresis (SDS-PAGE) and Zymogram

The molecular mass of the purified bacteriocin was estimated by Tricine SDS PAGE 16.5% gel and Zymogram [17,18]. The aliquot of purified bacteriocin and crude extract (CRE) were loaded in duplicate wells along with the Precision plus protein™ dual Xtra prestained protein standard (Bio-Rad Laboratories Inc., Hercules, CA, USA). The samples were electrophoresed in Tris-Tricine buffer solution at 10 mA for 4 h (Cleaver Scientific Ltd., Rugby, UK). The gel was divided so that each half contained both samples. One half-gel was stained with Coomassie blue, followed by a destaining step. Another half was used to detect the in-gel antimicrobial band by a Zymogram assay. It was fixed in a fixing solution (40% ethanol and 10% acetic acid) for 30 min, washed in water for 2 h, and placed onto a 7-mm layer of Mueller–Hinton agar (0.8% agar, 3% NaCl) inoculated with *V. parahaemolyticus* cells (~10^6^ CFU/mL). Another 7 mL of melting Mueller–Hinton ½ strength agar (0.8% agar, 3% NaCl) inoculated with *V*. *parahaemolyticus* cells was poured over the gel. After solidification, the plate was incubated for 8–12 h at 30 °C. An inhibition zone appeared in the gel if the samples contained bacteriocin.

### 2.9. Effects of Enzymes and Heat Treatment on Bacteriocin’s Activity

The antimicrobial spectrum of bacteriocin was estimated by well diffusion assay against reference strains, as mentioned above. The effects of heat treatment on the anti-*Vibrio* activity of bacteriocin were assessed by heating the aliquots of crude bacteriocin exact (CRE) at 60 °C, 80 °C, and 100 °C for 15 min, 30 min, and 60 min, respectively. The effects of enzymes (trypsin, pronase E, proteinase K, lysozyme) on the anti-*Vibrio* activity of bacteriocin were determined by treating CRE with 2 mg/mL of each enzyme at 37 °C for 1 h. All treated samples were tested for bioactivity against *V*. *parahaemolyticus* using a well diffusion assay and the untreated sample was used as a negative control.

## 3. Results

### 3.1. Antimicrobial Effect of Vi+

The evaluation of the antimicrobial activity of 16 isolated Vi+ was performed by an agar-based cross-streak assay. The results shown in Table 1 indicate that the majority of Vi+ display antimicrobial activity against a wide range of tested reference strains of both Gram-negative and Gram-positive bacteria, excepting two isolated Vi+, *B. flexus* NTCL3 and *B. cereus* NTCD2. The percentage of sensitive reference strains were found to be in the order of: *V*. *parahaemolyticus* (100%) > *V. harveyi* (93.8%) ≥ *B. cereus* (93.8%) > *L. monocytogenes* (87.5%) > *S. typhimurium* (62.5%) ≥ *E. coli* (50.0%) ≥ *S. aureus* (50.0%) > *C. albicans* (37.5%) > MRSA (0%). The intensity of activity against Gram-negative bacteria (*Vibrio* species, *Salmonella*, and *E. coli*) (+ to +++) appeared to be more significant than that against the Gram-positive bacteria (*B. cereus* or *S. aureus*). In addition, MRSA appeared to be resistant to the activity of Vi+, while *C. albicans* was only affected by intestinal Vi+.

After investigation of the relationship between antimicrobial activity and isolation sources, the intestinal Vi+ were found to produce more obvious antimicrobial activity against Gram-negative bacteria and yeasts (88.9% for *Salmonella*, 77.7% for *E. coli*, and 66.7% for *C. albicans*), higher than that of sponge-associated Vi+ (28.5% for *Salmonella*, 14.2% for *E. coli*, and 0% for *C. albicans*).

### 3.2. 16S rRNA Gene Analysis

All 16 isolated Vi+ were confirmed to be *Bacillus* via 16S rRNA gene sequencing analysis. Their nearest phylogenetic neighbor strains were presented in Table 2. A significant difference in species composition across two isolation sources is clearly observed. The intestinal Vi+ were abundant with members of the operational *B. amyloliquefaciens* subgroup (seven out of a total of nine Vi+), as well as *B. cereus* (1/9) and *B. flexus* (1/9), while the sponge-associated *Bacillus* species were mostly identified with members of the operational *B*. *pulmilus* subgroup (four out of a total of seven isolated Vi+), as well as *B. licheniformis* (2/7), and *B. amyloliquefaciens* (1/7). In this study, the operational *B. amyloliquefaciens* subgroup contains three *Bacillus* species (*B. amyloliquefaciens*, *B. velezensis*, and *B. methylotrophicus*). Similarly, the operational *B. pulmilus* subgroup contains two *Bacillus* species, *B. altitudinis* and *B*. *pulmilus*. This is because the 16S rRNA gene sequencing method is not so effective at distinguishing them [19,20].

During an inspection of the relationship between species and antimicrobial spectra, the majority of the *B. amyloliquefaciens* operational group showed dominant antimicrobial activity and strength when compared with other *Bacillus* species. The 16S rRNA sequences of all 16 Vi+ were deposited in GenBank with accession numbers MZ489228 to MZ489243.

### 3.3. PCR Amplification of PKS and NRPS Biosynthetic Genes

**Figure 2:**
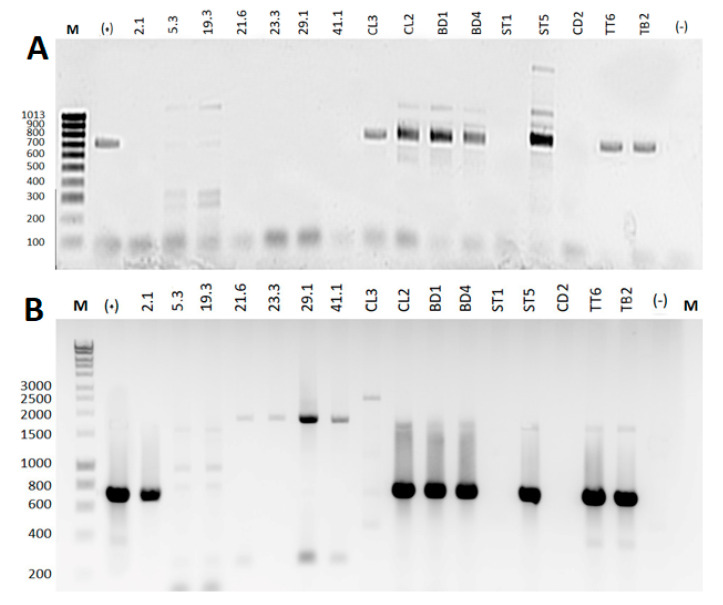
PCR amplification of Vi+ genomes for biosynthetic gene clusters of (**A**) NRPS using primer (A3F/A7R) and (**B**) PKS-I using primer (MDPQQR_f/HGTGT_r). Lanes, from left to right (M), included hyperLadder™ 100 bp (Bioline Reagents Limited, London, UK), positive control (+), followed by gene products amplified from seven sponge-associated Vi+ genomes (labeled with number code) and nine intestinal Vi+ genomes (labeled with letter–number code), and a negative control (-).

To explain the difference in antimicrobial spectra, all 16 Vi+ were used to check for the presence of NRPS, PKS-I, and PKS-II biosynthetic gene clusters in genomes via PCR amplification. The results are illustrated in Figure 2. About 50% of Vi+ showed positive amplification for at least one of two gene types (*NRPS* or *PKS--I*) but negative for the *PKS--II* gene. The results revealed that about 12.5% (2/16) Vi+ carried a single gene (*NRPS*, 1 strain; *PKS-I*, 1 strain), 33.3% of isolates (all intestinal Vi+) carried both genes, and 50.0% of isolates (sponge-associated Vi+, 6 strains; intestinal Vi+, 2 strains) were negative for all genes.

From an inspection of the relationship between sample types and antimicrobial genes and bacterial species, positive amplifications (66.7% for *PKS-I*, 66.7% for *NRPS*) were obtained for most intestinal Vi+, particularly members of the operational group *Bacillus amyloliquefaciens*, while the percentage of positive amplification for sponge-associated Vi+ (14.3% for *PKS-I*, 0% for *NRPS*) was significantly lower. In addition, the majority of the operational group *Bacillus amyloliquefaciens* contained both genes, while other *Bacillus* species (*B. pulmilus*, *B. licheniformis*, *B. flexus*, and *B. cereus*) lacked these antimicrobial genes or carried only one targeted gene.

### 3.4. Sequencing and Sequencing Analysis of (PKS-I) Gene Products

The gene product (PKS-I), which was amplified from the PQBB2.1 isolate using a primer (MDPQQR_f/HGTGT_r), was sequenced. The sequence analysis using BLASTx at NCBI showed the amino acid sequence of the PKS-I gene product, which possessed 95.5% identity with ketosynthase of *Bacillus* species (accession no. AGO59067.1) or 94.0% identity with polyketide synthase type I PksL of *Bacillus amyloliquefaciens* (accession no. AOC91145.1), for confirmation of the amplified product.

### 3.5. A Three-Step Procedure for the Rapid Purification of Bacteriocin-Like Substance from Bacterial Cells

**Figure 3:**
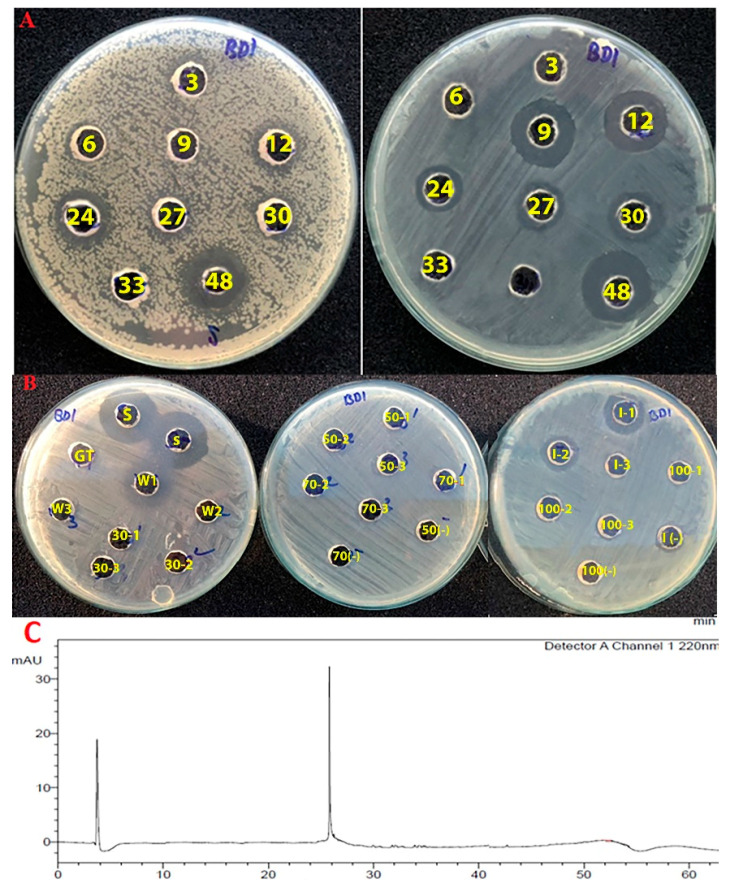
Purification of the anti-*Vibrio* bacteriocin produced by *B. methylotrophicus* NTBD1. (**A**) Anti-*Vibrio* activity (left) and anti-*Listeria* activity (right) of the bacterial cultures obtained by different cultivation times. Wells (3)–(48) reflect cultures collected after 3–48 h, respectively. (**B**) Purification of bacteriocin crude extract by hydrophobic-interaction chromatography. Anti-*Vibrio* activity of crude extract (S), crude extract after loading through column (GT), the column-wash with 2% methanol (0.1% TFA) (W), column-elutes with 30% (30), 50% (50), 70% (70), 70% isopropanol (0.1% TFA) (I), and 100% of methanol (0.1% TFA) (100), respectively. Each step was conducted with three column volumes (code (1–3) follows each solvent concentration), with each solvent used as a negative control (–). The results indicated that the column eluted with 70% isopropanol (I) showed anti-*Vibrio* activity. (**C**) Purification of this active fraction (I) by reverse-phase HPLC resulted in a bacteriocin peak at a retention time of 25.6 min, monitored only with a UV wavelength of 220 nm.

The effect of cultivation time on the antimicrobial production of *B. methylotrophicus* NTBD1 was estimated first. Figure 3A shows that anti-*Vibrio* activity was initially observed in cultures at 12 h (11 mm), increased at 24 h (12 mm), decreased afterward, and appeared at 48 h with the largest killing diameter (20 mm). Meanwhile, the anti-*Listeria* activity of the culture was observed at an earlier cultivation time of 9 h (18 mm), maintained activity at 12 h (18 mm) but significantly reduced afterward, and appeared again at 48 h with the largest killing diameter (21 mm). This indicates that multiple antimicrobial compounds are produced by this bacterium. The killing diameter above includes the diameter of the well (6 mm). However, proteolytic treatment of active cultures with pronase E showed that the anti-*Vibrio* activity of the 24-h-old culture was completely lost after the proteolysis, while the bioactivity of the 36-h-old culture was resistant to the proteolysis. Therefore, the 24-h-old culture was selected for the purification of bacteriocin from this bacterium.

*B. methylotrophicus* NTBD1 was cultured at 30 °C in 1 L LMPB for 24 h, then the cells were collected after centrifugation at 4500× *g* for 20 min. A three-step procedure was applied to purify the bacteriocin binding on the bacterial cells, including: (i) preparation of a crude bacteriocin extract (CRE) by stirring cells in 70% isopropanol (pH 2) to release the bacteriocin, following by removal of the solvent; (ii) partial purification of CRE by hydrophobic interaction chromatography (HIC) using a C18 cartridge (Waters Corporation, Milford, MA, USA); and (iii) purification of the HIC-based active fraction by reverse-phase HPLC to collect the bacteriocin. Figure 3B demonstrates the anti-*Vibrio* activities of fractions obtained during hydrophobic interaction chromatography. The results show that only the column eluted with 70% isopropanol (designed as IPA) exhibited anti-*Vibrio* activity. Subsequent purification of this concentrated IPA fraction by some cycles of reverse-phase HPLC obtained a single bacteriocin peptide with retention time at 25.6 min (Figure 3C). In detail, elutes in the first cycle were collected at intervals of 2 min, and the anti-*Vibrio* fraction was obtained at a retention time of 24–26 min. This active fraction was concentrated and introduced to RP-HPLC cycles until we obtained a single peak of bacteriocin; elutes were obtained by the peak-picking method.

### 3.6. The Thermal and Antimicrobial Properties of the Bacteriocin

**Figure 4:**
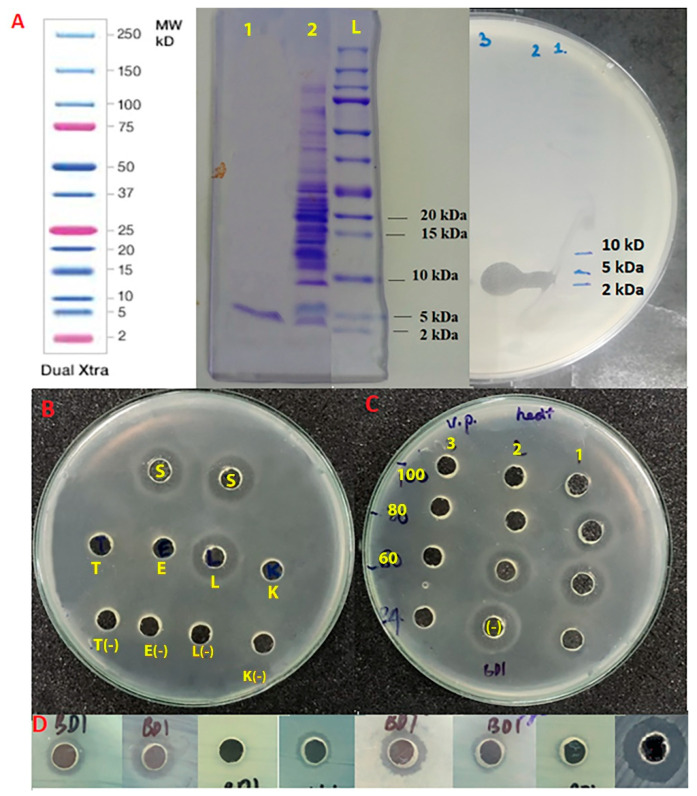
Properties of bacteriocin. (**A**) Molecular weight estimated by Tricine SDS-PAGE 16.5% gel (left) and Zymogram gel (right). Position of wells on gels included HPLC-based bacteriocin (1), bacteriocin crude extract (2), protein ladder (L). (**B**) Anti-*Vibrio* activity of bacteriocin after proteolytic treatment with pronase E (E), proteinase K (K), trypsin (T), and lysozyme (L). (**C**) Anti-*Vibrio* activity of bacteriocin after thermal treatment at 60 °C (60), 80 °C (80), 100 °C (100) for 15 min (1), 30 min (2), and 60 min (3). (**D**) The antimicrobial activity of bacteriocin evaluated by a well diffusion assay. The reference strains in the images (from left to right) included *S. typhimurium*, V. *parahaemolyticus*, *E. coli*, *V. harveyi*, *B*. *cereus*, *S. aureus*, MRSA, and *L. monocytogenes*.

The aliquots of the HPLC-based bacteriocin and crude extract were loaded on Tricine SDS-PAGE 16.5% gel. The results of both stained gel and Zymogram gel (Figure 4A) indicated that two samples contained only an anti-*Vibrio* substance with a molecular weight of 2–5 kDa.

In addition, the effects of enzymes, thermal treatment, and the antimicrobial spectrum of bacteriocin were evaluated and the results are given in Figure 4B–D, respectively. In these assays, crude extract of bacteriocin (CRE) was used to replace the purified bacteriocin due to the limited quantity of purified bacteriocin and possessing the same antimicrobial substance. The result of the enzymatic digestion assay (Figure 4B) shows that the anti-*Vibrio* activity of bacteriocin was completely eliminated after proteolytic treatments using proteinase K, pronase E, and trypsin enzymes but not lysozyme. Compared with unheated crude extract, the anti-*Vibrio* activity of bacteriocin was still active after heating at 60 °C for up to 30 min and reduced after thermal treatment at 80 °C for 15 min, but completely inactive after treatments at 100 °C for 15, 30, and 60 min. Heat stability is essential to apply bacteriocin as a feed additive. In fact, heat treatment commonly plays an essential role in many manufacturing stages. Furthermore, the analysis indicated that the bacteriocin displayed antimicrobial activity against a wide range of both Gram-negative (*S*. *typhimurium*, *V. parahaemolyticus*, and *V. harveyi*) and Gram-positive bacteria (*B*. *cereus*, *L. monocytogenes*, and *S. aureus*), but not against *E. coli* or MRSA.

## 4. Discussion

Vibriosis in farmed aquatic animals causes financial losses. There is increasing interest in using probiotics in aquaculture for the prevention of *Vibrio* infection. Therefore, this study aimed to characterize 16 anti-*Vibrio* probiotics isolated from two isolation sources, sponge and fish intestines collected in Vietnam, and one anti-*Vibrio* bacteriocin, for later use in aquaculture.

For the analysis of 16S rRNA, the identification of all 16 Vi+ as *Bacillus* species is surprising given that other Vi+ species (*Vibrio*, *Proteus*, and *Streptomyces*) are absent from the list [5,8]. This raises the question of whether *Bacillus* species are the main natural probiotics for aquatic animals against enteropathogenic pathogens in these regions [21,22,23]. The distribution of dominant *Bacillus* species may be due to their particular advantages. With the production of diversely structural antimicrobials, *Bacillus* species can colonize a wide range of marine samples and limit the growth of many counterproductive bacteria. In terms of safety, *Bacillus* species are optimal probiotic strains compared with other Gram-negative Vi+ (*Proteus* or *Vibrio*) used as probiotics. Gram-negative bacteria can serve as vectors for the transmission of toxins or resistant genes on aquaculture farms. In addition, this study provides the first identification of Vi+ strains from marine sponges collected in the Vietnam Sea, which has not yet been studied.

Vi+ are likely to display the same pattern of antimicrobial properties. They have a broad spectrum of responses toward bacteria, both Gram-negative and Gram-positive, but exhibit significantly higher efficacy against Gram-negative bacteria (*Vibrio* sp., *Salmonella*, *E. coli*) compared with Gram-positive bacteria (*Staphylococcus*, *B. cereus*, and MRSA). In particular, the antibacterial property of intestinal Vi+ is clearly observed. This can be supposed to be a typical activity of Vi+ because the property is similar to that of previously isolated Vi+ but in contrast with the majority of conventional *Bacillus.* For instance, two intestinal Vi+, *B. subtilis* G024 and *B. amyloliquefaciens* N004, also displayed high efficacy against a wide range of Gram-negative bacteria (*V. anguillarum*, *V. campbellii*, *V. vulnificus*, *V. parahaemolyticus*, and *E. tarda*), but only against one Gram-positive bacterium (*S. aureus* or *B. cereus*) [24]. Two other intestinal Vi+, *B. velezensis* TPS3N, and *B. amyloliquefaciens* TPS17, showed strong activity against *A. hydrophila* and *V. harveyi,* but inhibited *S. agalactiae* with only limited strength [25]. On the other hand, a *Bacillus* sp. SW1-1 inhibited the growth of many Gram-negative bacteria (*E. tarda*, *V. anguillarum*, and *V. harveyi*), and *Streptococcus* species [26]. In contrast, conventional *Bacillus* sp. strains frequently have a higher antimicrobial efficacy toward Gram-positive bacteria than Gram-negative bacteria. For instance, more than 83% of marine *Bacillus* isolated from marine samples (sponges, seaweeds) in the Nha Trang sea region showed remarkable activity toward Gram-positive bacteria (*B. cereus*, *S. aureus*, MRSA, *Clostridium*, and *Streptococcus sp.*) [27]. Additionally, many *Bacillus* sp. strains isolated from the soil sample were reported to have better effects against Gram-positive bacteria compared to Gram-negative bacteria [28]. Many *Bacillus* species produce a large number of antimicrobial peptides that only affect Gram-positive bacteria [9]. Thus, *Bacillus*-derived antimicrobial peptides (PKS, NRPS, bacteriocins) are commonly cationic compounds and promote binding onto the outer membranes of Gram-positive bacteria for disruption of the membrane biosynthesis [9,29]. In addition, all reference strains in this study were selected to represent current foodborne pathogens (*L. monocytogenes*) or livestock/human diseases (*Salmonella*, *E. coli*, and *B. cereus*). Therefore, the results emphasize the great potential of these Vi+ for uses in multiple fields (aquaculture, veterinary, medical, and the food industry).

To explain the difference in antimicrobial properties, biosynthetic gene clusters of non-ribosomal peptide (NRPS), polyketide type I (PKS-I), and polyketide type II (PKS-II) were amplified from Vi+ genomes. The results indicate that the difference in antimicrobial spectra between Vi+ and conventional *Bacillus* is associated with the higher frequency of *PKS-I* and *NRPS* genes in Vi+. Approximately 50% of Vi+ were found to carry at least one of two gene types (43.8% for *NRPS* and 43.8% for *PKS-I* genes), but not *PKS-II*. Meanwhile, a recent whole-genome mining study estimated that a lower percentage (~31%) of Firmicutes carried *NRPS* and *PKS* gene clusters [30]. In a recent study, Mien et al. (2020) also reported a lower percentage for *NRPS* and *PKS-I* genes (24.0% and 0%, respectively), but higher for *PKS-II* genes (20.0%), after PCR amplification of 25 marine bacteria isolated from the Central Sea region of Vietnam [31]. Similarly, a lower percentage of *NRPS* gene (38%) and *PKS-I* gene (38%) but a higher percentage of *PKS-II* (54%) were observed among 50 invertebrate-associated bacteria from the Red Sea [15]. The absence of the *PKS-II* gene in Vi+ conflicts with observations from earlier studies. This is possibly due to the present work omitting Actinobacteria, one of the rich sources of diversely structural polyketide type II [32].

In addition, intestinal Vi+ produced more obvious activity against Gram-negative bacteria and yeast than sponge-associated Vi+. This is possibly due to the higher frequency of *PKS-I* and *NRPS* genes from intestinal Vi+ (66.7%) in comparison with sponge-associated Vi+ genomes (0% and 14.3%). The difference can be derived from different strain variants across the two sample types. Thus, intestinal Vi+ were mostly identified as operational group *B. amyloliquefaciens* because the majority of them carried both antimicrobial genes, while sponge-associated Vi+ were identified as mostly members of operational group *B. pulmilus* and *B. licheniformis*, carrying no such genes or fewer genes in general. This raises the question of whether these *PKS-I* and *NRPS* genes are constrained to intestinal *B. amyloliquefaciens* in these sea regions. The findings also emphasize the potential of operational group *B. amyloliquefaciens* to contain the most promising probiotics for aquaculture use. The absence of antimicrobial genes from sponge-associated Vi+ may indicate the possible production of other antimicrobial substances (bacteriocin) because *Bacillus* species are well known as producers of both anti-*Vibrio* bacteriocins and non-ribosomally synthesized peptides [33]. However, PCR amplification of bacteriocin genes from these Vi+ was not performed in this study due to the diversity of bacteriocin associated with *Bacillus* species [34]. We suggest further study for the discovery of bacteriocins from these *Bacillus* Vi+ for their applications.

In contrast with non-ribosomal peptides and polyketides, which play more important roles in agriculture and aquaculture, bacteriocins can potentially serve as promising feed additives or seafood preservatives. Bacteriocin can be delivered into the guts of aquatic animals via feeds for rapid treatment of *Vibrio* pathogens. Therefore, an effort was made to purify a bacteriocin-like substance produced by *B. methylotrophicus* NTBD1 for a study on their characterization. Bacteriocin from intestinal Vi+ may ensure activity under a gut condition in comparison with that from sponge-associated Vi+. A three-step procedure was performed for the rapid recovery of bacteriocin from bacterial cells, as reported earlier [16]. The presence of bacteriocin on the bacterial cell surface may be due to interactions between cationic bacteriocin and negatively charged cell membrane components. It is found that the bacterium can produce multiple anti-*Vibrio* substances, one at 24 h (supposed to be bacteriocin due to its proteolytic sensitiveness) and others at 48 h (supposed to be non-ribosomally synthesized peptides due to their proteolytic resistance and production at the end of the stationary phase). In the literature, a *B. amyloliquefaciens* FZB42 also produced five different antimicrobial peptides, including two bacteriocins and three non-ribosomally synthesized peptides [34]. For the study on the characterizations, the purified bacteriocin produced by *B. methylotrophicus* NTBD1 had a molecular weight of ~3–5 kDa, high thermal stability, and good sensitivity to protease enzymes, and exhibited broad antibacterial ability against both Gram-negative and Gram-positive bacteria. As members of the *B. amyloliquefaciens* operational group, these characteristics of bacteriocin produced by *B. methylotrophicus* NTBD1 are similar to the majority of other *Bacillus amyloliquefaciens*-derived bacteriocins. For instance, the intestinal *Bacillus* strain NM12 produced an anti-*Vibrio* bacteriocin that was unstable in heat, had a low molecular mass (less than 5 kDa), and showed broad antibacterial activity toward many human and eel pathogens including *V*. *vulnificus* [4]. Bacteriocin BaCf3, produced by the intestinal *B. amyloliquefaciens* BTSS3, possesses broad antimicrobial activity, thermal stability, and pH tolerance [7]. In case of bacteriocin being used as a feed additive, all of these characteristics are essential to ensure the stability of bacteriocin activity under heat treatment, which commonly plays an essential role in many manufacturing stages. In addition, the broad antimicrobial spectrum of bacteriocin suggests the potential applications of bacteriocin, not only in aquaculture but also for the prevention of food spoilage bacteria like *Listeria monocytogenes* or *Salmonella* species in the food industry.

## 5. Conclusions

The Vi+ associated with the intestines of aquatic animals and sponges collected from the Vietnam Sea show high biodiversity and broad distribution of dominant *Bacillus* species, but with different strain variants across two sample types. These Vi+ are likely to possess typical antimicrobial properties compared with conventional *Bacillus*, which has a broad spectrum of activity against wide panels of the current aquaculture, foodborne, and clinically relevant pathogens but with significant antimicrobial efficacy against Gram-negative bacteria rather than Gram-positive bacteria. In addition, differences in the antimicrobial spectrum and species composition are observed from Vi+ strains across the two sample types, with obvious antimicrobial activity of the intestinal *Bacillus* rather than the sponge-associated Vi+. This study emphasizes the probiotic potential of intestinal *Bacillus*, particularly members of the operational *B. amyloliquefaciens* subgroup, for later use in sustainable aquaculture and other fields. The presence of a higher frequency of the biosynthetic gene clusters of PKS or NRPS or both in intestinal Vi+ (77.8%) than in the sponge-associated Vi+ (14.3%) can explain the difference. In addition, not only do intestinal Vi+ exhibit great potential as live probiotics in aquaculture farming but also bacteriocin possesses the necessary physicochemical properties for later use as a promising feed additive. However, all applications require further trials before these probiotic Vi+ can be used in practical treatment.

## Figures and Tables

**Table 1:** Antimicrobial activity of 16 marine isolated Vi+ against foodborne and animal pathogens including *V. harveyi* (*Vh*), *V. parahaemolyticus* (Vp), *E. coli* (Ec), *S. typhimurium* (Sa), *S. aureus* (Stp), methicillin-resistant *Staphylococcus aureus* (MRSA), *B. cereus* (Bc), *L. monocytogenes* (Li), *C. albicans* (Ca). (-) No activity, (+) to (+++) reflects increasing bioactivity.

No	Isolates	Reference Strains								
		Vh	Vp	Sa	Ec	Bc	Stp	Li	Ca	MRSA
	*Sponge-associated isolated Vi+*									
1	PQBB2.1	-	++	-	-	+	-	++	-	-
2	PQBB5.3	+++	+++	+	-	+	-	++	-	-
3	PQBB19.3	+	+	-	-	+	+	-	-	-
4	PQBB21.6	++	++	-	-	++	-	++	-	-
5	PQBB23.3	+++	++	-	-	+	-	++	-	-
6	PQBB29.1	+++	++	-	-	+	-	++	-	-
7	PQBB41.1	+++	+++	+	+	+	-	++	-	-
Killing percent (%)		85.7	100	28.5	14.2	100	14.2	85.7	0	0
	*Intestinal isolated* Vi+									
8	NTCL2	+++	+++	++	+	++	++	++	++	-
9	NTCL3	+	+	-	-	-	-	-	-	-
10	NTBD1	++	++	++	+	+	+	++	+	-
11	NTBD4	+++	+++	+	+	+	+	++	+	-
12	NTST1	+++	+++	++	+	++	++	++	-	-
13	NTST5	+++	++	+	+	+	+	++	+	-
14	NTCD2	++	++	-	-	+	+	++	+	-
15	NTTB2	++	++	+	+	+	+	++	-	-
16	NTTT6	+++	++	++	+	+	+	++	++	-
Killing percent (%)		100	100	77.7	77.7	88.9	88.9	88.9	66.7	0
Killing percent by all Vi+ (%)		93.8	100	62.5	50.0	93.8	50.0	87.5	37.5	0

**Table 2:** Sequence analysis of 16S rRNA genes of the Vi+ using BLAST n.

	Code	Accession Number	Name of the Most Closely Related Strains	Maximum Score	Identity (%)	Accession Number
*Sponge-associated Vi+ isolates*						
1	PQBB2.1	MZ489228	*Bacillus amyloliquefaciens* MPA	2128	97.4	NR_117946.1
2	PQBB5.3	MZ489229	*Bacillus licheniformis* DSM 13	1842	99.0	NR_118996.1
3	PQBB19.3	MZ489230	*Bacillus licheniformis* 302-2	1869	98.5	MT795776.1
4	PQBB21.6	MZ489231	*Bacillus pumilus* TBMAX76	1618	99.2	MK834714.1
5	PQBB23.3	MZ489232	*Bacillus pumilus* ChST1.7	1663	98.4	JF935095.1
6	PQBB29.1	MZ489233	*Bacillus pumilus* CBS-i1	2121	99.4	GQ220330.1
7	PQBB41.1	MZ489234	*Bacillus altitudinis* NPB34b	2015	98.4	MT598007.1
*Intestinal Vi+ isolates*						
8	NTCL2	MZ489235	*Bacillus velezensis* InAD-161	1941	97.7	KY859772.1
9	NTCL3	MZ489236	*Bacillus flexus strain* LE9	1917	99.3	MT279468.1
10	NTBD1	MZ489237	*Bacillus methylotrophicus* S611Ba-40	1386	98.2	HQ238543.1
11	NTBD4	MZ489238	*Bacillus amyloliquefaciens* DH8030	2228	99.1	CP041770.1
12	NTST1	MZ489239	*Bacillus amyloliquefaciens* P1	2176	98.2	MT416658.1
13	NTST5	MZ489240	*Bacillus velezensis* BRM 046306	2156	98.1	MK461867.1
14	NTCD2	MZ489241	*Bacillus cereus* LA333	1816	98.2	KY622412.1
15	NTTT6	MZ489242	*Bacillus amyloliquefaciens* W36	2115	99.1	MN922613.1
16	NTTB2	MZ489243	*Bacillus. velezensis* InAD-161	1941	97.8	KY859772.1

## Data Availability

Data can be shared upon request.

## References

[1] Thompson F.L., Iida T., Swings J. (2004). Biodiversity of vibrios. Microbiol. Mol. Biol. Rev..

[2] Hong To T.T., Yanagawa H., Khanh Thuan N., Hiep D.M., Cuong D.V., Khai L.T.L., Taniguchi T., Kubo R., Hayashidani H. (2020). Prevalence of *Vibrio parahaemolyticus* causing acute hepatopancreatic necrosis disease of shrimp in shrimp, molluscan shellfish and water samples in the Mekong delta, Vietnam. Biology.

[3] Kewcharoen W., Srisapoome P. (2019). Probiotic effects of *Bacillus* spp. from Pacific white shrimp (*Litopenaeus vannamei*) on water quality and shrimp growth, immune responses, and resistance to *Vibrio parahaemolyticus* (AHPND strains). Fish. Shellfish. Immunol..

[4] Sugita H., Hirose Y., Matsuo N., Deguchi Y. (1998). Production of the antibacterial substance by *Bacillus* sp. strain NM 12, an intestinal bacterium of Japanese coastal fish. Aquaculture.

[5] Nguyen V.D., Pham T.T., Nguyen T.H., Nguyen T.T., Hoj L. (2014). Screening of marine bacteria with bacteriocin-like activities and probiotic potential for ornate spiny lobster (*Panulirus ornatus*) juveniles. Fish. Shellfish. Immunol..

[6] Xu H.M., Rong Y.J., Zhao M.X., Song B., Chi Z.M. (2014). Antibacterial activity of the lipopetides produced by *Bacillus amyloliquefaciens* M1 against multidrug-resistant *Vibrio* spp. isolated from diseased marine animals. Appl. Microbiol. Biotechnol..

[7] Bindiya E.S., Tina K.J., Sasidharan R.S., Bhat S.G. (2019). BaCf3: Highly thermostable bacteriocin from *Bacillus amyloliquefaciens* BTSS3 antagonistic on food-borne pathogens. 3 Biotech.

[8] Carraturo A., Raieta K., Ottaviani D., Russo G.L. (2006). Inhibition of *Vibrio parahaemolyticus* by a bacteriocin-like inhibitory substance (BLIS) produced by *Vibrio mediterranei* 1. J. Appl. Microbiol..

[9] Sumi C.D., Yang B.W., Yeo I.C., Hahm Y.T. (2015). Antimicrobial peptides of the genus *Bacillus*: A new era for antibiotics. Can. J. Microbiol..

[10] Wahyudi A.T., Priyanto J.A., Maharsiwi W., Astuti R.I. (2018). Screening and characterization of sponge-associated bacteria producing bioactive compounds anti-*Vibrio* sp.. Am. J. Biochem. Biotechnol..

[11] Lertcanawanichakul M., Sawangnop S. (2008). A comparison of two methods used for measuring the antagonistic activity of *Bacillus* species. Walailak. J. Sci. Tech..

[12] Srinivasan R., Karaoz U., Volegova M., MacKichan J., Kato-Maeda M., Miller S., Nadarajan R., Brodie E.L., Lynch S.V. (2015). Use of 16S rRNA gene for identification of a broad range of clinically relevant bacterial pathogens. PLoS ONE.

[13] Liu J., Li F., Kim E.L., Li J.L., Hong J., Bae K.S., Chung H.Y., Kim H.S., Jung J.H. (2011). Antibacterial polyketides from the jellyfish-derived fungus *Paecilomyces variotii*. J. Nat. Prod..

[14] Setiaji J., Feliatra F., Teruna H.Y., Lukistyowati I., Suharman I., Muchlisin Z.A., Johan T.I. (2020). Antibacterial activity in secondary metabolite extracts of heterotrophic bacteria against *Vibrio alginolyticus, Aeromonas hydrophila*, and *Pseudomonas aeruginosa*. F1000Research.

[15] El Samak M., Solyman S.M., Hanora A. (2018). Antimicrobial activity of bacteria isolated from Red Sea marine invertebrates. Biotechnol. Rep..

[16] Busarcevic M., Dalgalarrondo M. (2012). Purification and genetic characterisation of the novel bacteriocin LS2 produced by the human oral strain *Lactobacillus salivarius* BGHO1. Int. J. Antimicrob. Agents..

[17] Akter N., Hashim R., Pham H.Q., Choi S.D., Lee D.W., Shin J.H., Rajagopal K. (2020). *Lactobacillus acidophilus* antimicrobial peptide Is antagonistic to *Aeromonas hydrophila*. Front. Microbiol..

[18] Schägger H. (2006). Tricine-SDS-PAGE. Nat. Protoc..

[19] Fan B., Blom J., Klenk H.P., Borriss R. (2017). *Bacillus amyloliquefaciens, Bacillus velezensis, and Bacillus siamensis* form an “operational group *B. amyloliquefaciens*” within the *B. subtilis* species complex. Front. Microbiol..

[20] Liu Y., Lai Q., Dong C., Sun F., Wang L., Li G., Shao Z. (2013). Phylogenetic diversity of the *Bacillus pumilus* group and the marine ecotype revealed by multilocus sequence analysis. PLoS ONE.

[21] Thankappan B., Ramesh D., Ramkumar S., Natarajaseenivasan K., Anbarasu K. (2015). Characterization of *Bacillus* spp. from the gastrointestinal tract of *Labeo rohita*-towards to identify novel probiotics against fish pathogens. Appl. Biochem. Biotechnol..

[22] Santos R., André C., Oliva-Teles A., Saavedra M., Enes P., Serra C. (2016). Fish gut sporeformers to control fish pathogens. Front. Mar. Sci..

[23] Kavitha M., Raja M., Perumal P. (2018). Evaluation of probiotic potential of *Bacillus* spp. isolated from the digestive tract of freshwater fish *Labeo calbasu* (Hamilton, 1822). Aquac. Rep..

[24] Chen Y., Li J., Xiao P., Zhu W., Mo Z. (2016). The ability of marine *Bacillus* spp. isolated from fish gastrointestinal tract and culture pond sediment to inhibit growth of aquatic pathogenic bacteria. Iran. J. Fish. Sci..

[25] Kuebutornye F.K.A., Lu Y., Abarike E.D., Wang Z., Li Y., Sakyi M.E. (2020). In vitro assessment of the probiotic characteristics of three *Bacillus* species from the gut of Nile Tilapia, *Oreochromis niloticus*. Probiotics. Antimicrob. Proteins..

[26] Kim Y.-O., Park I., Kim D.J., Nam B., Kim D.-G., Jee Y., An C. (2014). Identification and characterization of a bacteriocin produced by an isolated *Bacillus* sp. SW1-1 that exhibits antibacterial activity against fish pathogens. J. Korean Soc. Appl. Biol. Chem..

[27] Chau K.M., Van Quyen D., Fraser J.M., Smith A.T., Van T.T.H., Moore R.J. (2020). Broad spectrum antimicrobial activities from spore-forming bacteria isolated from the Vietnam Sea. PeerJ.

[28] Yilmaz M., Soran H., Beyatli Y. (2006). Antimicrobial activities of some *Bacillus* spp. strains isolated from the soil. Microbiol. Res..

[29] Omardien S., Brul S., Zaat S.A. (2016). antimicrobial activity of cationic antimicrobial peptides against Gram-positives: Current progress made in understanding the mode of action and the response of bacteria. Front. Cell. Dev. Biol..

[30] Wang H., Fewer D.P., Holm L., Rouhiainen L., Sivonen K. (2014). Atlas of nonribosomal peptide and polyketide biosynthetic pathways reveals common occurrence of nonmodular enzymes. Proc. Natl. Acad. Sci. USA.

[31] Mien P.T., Ha D.V., Ben H.X., Chen B., Liu L., Minh-Thu P. (2020). Antimicrobial activities of sponge-derived microorganisms from coastal waters of central Vietnam. J. Mar. Sci. Eng..

[32] Selvin J., Sathiyanarayanan G., Lipton A.N., Al-Dhabi N.A., Valan Arasu M., Kiran G.S. (2016). Ketide synthase (KS) domain prediction and analysis of iterative type II PKS gene in marine sponge-associated Actinobacteria producing biosurfactants and antimicrobial agents. Front. Microbiol..

[33] Abriouel H., Franz C.M., Ben Omar N., Gálvez A. (2011). Diversity and applications of *Bacillus* bacteriocins. FEMS. Microbiol. Rev..

[34] Fan B., Wang C., Song X., Ding X., Wu L., Wu H., Gao X., Borriss R. (2018). *Bacillus velezensis* FZB42 in 2018: The gram-positive model strain for plant growth promotion and biocontrol. Front. Microbiol..

